# CDS-BART: a BART-based foundation model for mRNA sequence analysis

**DOI:** 10.1093/bioadv/vbag259

**Published:** 2026-09-15

**Authors:** Erkhembayar Jadamba, Sang-Heon Lee, Sungho Lee, Hyekyoung Lee, Jinhee Hong, Hyunjin Shin

**Affiliations:** MOGAM Institute for Biomedical Research, Seoul, 06730, Republic of Korea; MOGAM Institute for Biomedical Research, Seoul, 06730, Republic of Korea; MOGAM Institute for Biomedical Research, Seoul, 06730, Republic of Korea; MOGAM Institute for Biomedical Research, Seoul, 06730, Republic of Korea; MOGAM Institute for Biomedical Research, Seoul, 06730, Republic of Korea; MOGAM Institute for Biomedical Research, Seoul, 06730, Republic of Korea

## Abstract

**Summary:**

Recent advancements in artificial intelligence (AI) have led to the development of foundation models that interpret mRNA as a language. Notable examples include CodonBERT, HydraRNA, Evo 2, and Helix-mRNA. These models demonstrate significant potential as powerful tools for mRNA research. However, to the best of our knowledge, there is currently no publicly available AI model that is both easy to use and capable of analysing mRNA sequences up to about 4 kb, a length scale typical of many therapeutic mRNAs, including those encapsulated within lipid nanoparticles. Thus, we propose CDS-BART, a user-friendly, open-source tool that integrates SentencePiece subword tokenization with the denoising sequence-to-sequence training of Bidirectional and Auto-Regressive Transformers (BART). CDS-BART was pre-trained on mRNA data from nine taxonomic groups provided by the NCBI RefSeq database. This comprehensive pre-training, coupled with BART’s denoising capability, supports learning of coding sequence (CDS)-level sequence regularities that are useful for downstream mRNA property prediction. Thus, CDS-BART can ultimately deliver robust performance across a wide range of mRNA prediction tasks.

**Availability and implementation:**

CDS-BART is released under the MIT License. Latest code is available via GitHub at https://github.com/mogam-ai/CDS-BART and archived on Zenodo (DOI: 10.5281/zenodo.21502775).

## 1 Introduction

Over the last few years, mRNA-based vaccines and therapeutics have become a major focus in new drug development (Pardi and Krammer 2024). Traditionally, bioinformatics-based algorithms like RNAfold (Lorenz *et al.* 2011) or LinearPartition Zhang *et al.* (2020) have been used for RNA research; however, these methods are only feasible for a limited range of downstream tasks such as mRNA secondary structure and property predictions. To overcome this limitation, large language models (LLMs) including Bidirectional Encoder Representations from Transformers (BERT), Bidirectional and Auto-Regressive Transformers (BART), and Generative Pre-trained Transformers (GPT) have inspired an approach that handles mRNA as a linguistic system with complex patterns and contextual rules. These language-based AI models serve as foundation models that enable researchers to conduct a wide range of tasks simultaneously, such as mRNA structure prediction and sequence generation (Li *et al.* 2024, 2025a, Brixi *et al.* 2026).

These mRNA language models exhibit distinctive strengths but also have limitations. For example, depending on their architectures, some LLMs struggle to process long mRNA sequences efficiently. Human mRNA coding sequences (CDSs) average about 1.8 kb in length, and fewer than 5% exceed 4 kb (Supplementary Methods, Data processing). Non-replicating mRNA vaccines encode only the target antigen and are about 2–3 kb long (Zhang *et al.* 2025). Even the widely used COVID-19 vaccines BNT162b2 and mRNA-1273 deliver lipid-nanoparticle (LNP)-encapsulated mRNAs of roughly 4 kb (Silva-Pilipich *et al.* 2024). Similarly, gene-editing therapeutics provide a comparable benchmark, and the Streptococcus pyogenes Cas9 CDS is about 4.1 kb (Xiong *et al.* 2016). Therefore, a practical 4 kb upper bound covers most mRNAs used in vaccines and genome-editing.

While BERT’s masked language modeling facilitates fine-tuning for numerous downstream tasks, it is less suited for direct sequence-to-sequence (seq2seq) transformations and the analysis of very long input sequences (Devlin *et al.* 2019). CodonBERT attempts to address this by tokenizing nucleotide triplets (Li *et al.* 2024), thus extending the input sequence length limit. However, transcripts longer than approximately 3 kb still remain out of reach. To tackle these limitations, mRNA-LM (Li *et al.* 2025b), HydraRNA (Li *et al.* 2025a), Helix-mRNA (Wood *et al.* 2025), and Evo 2 (Brixi *et al.* 2026) have been proposed more recently. Among them, mRNA-LM allows the user to conduct more comprehensive analysis on 5′ UTR, coding sequences (CDS), and 3′ UTR together through contrastive language-image pre-training (CLIP) (Li *et al.* 2025b). On the other hand, HydraRNA and Helix-mRNA employ a bidirectional state-space model (SSM) with or without multi-head attention for full-length RNA coverage (Li *et al.* 2025a, Wood *et al.* 2025). Another approach, Evo 2, uses a convolutional multi-hybrid architecture, called StripedHyena2, to merge local and global contexts efficiently (Brixi *et al.* 2026). However, models relying on SSM or StripedHyena2 can be complex and computationally expensive, limiting their accessibility, developability, and ease of training.

In our study, we addressed these challenges by developing CDS-BART, a new mRNA foundation model that integrates SentencePiece (Kudo and Richardson 2018) and BART (Lewis *et al.* 2020). SentencePiece is a data-driven subword tokenization method that can represent genomic sequences using fewer non-overlapping variable-length tokens (Zhou *et al.* 2024). As a result, CDS-BART can process mRNA sequences up to about 4 kb in length as input, aligning with the upper payload limit of current LNP formulations. In addition, BART’s seq2seq learning setup and denoising pre-training allow the model to take a noisy or incomplete input sequence, encode its meaning, and then generate a reconstructed input sequence that meets the task goal, unlike BERT’s token-level fill-in approach. Furthermore, our model was trained on mRNA sequence data provided by the NCBI RefSeq database, including sequences of nine taxonomic groups. This diversity of the training data ensures broad coverage of complex biological patterns. In conclusion, we strongly believe that CDS-BART will serve as a practical foundation model for effective mRNA sequence property prediction, optimization, and design, particularly in the development of mRNA-based vaccines or therapeutics.

## 2 Methods

### 2.1 Overview of the CDS-BART framework

CDS-BART is a framework for CDS-based mRNA sequence modeling, as illustrated in Fig. 1. The overall workflow consists of four steps: construction of a large-scale CDS pre-training corpus, CDS-specific tokenization, transformer-based pre-training, and supervised fine-tuning for downstream mRNA property prediction. Given a CDS sequence, CDS-BART first applies CDS-specific filtering and converts the nucleotide sequence into variable-length tokens using a SentencePiece-based BPE tokenizer (Fig. 1A and B). The tokenized CDS sequences are then used for pre-training, during which we compared alternative transformer-based objectives and selected the denoising variant as the final CDS-BART model for downstream evaluation. After pre-training, CDS-BART is fine-tuned using sequence-level regression or classification objectives. We evaluated the model on six CodonBERT-derived benchmark datasets covering protein expression, gene expression, mRNA stability, riboswitch activity, and RNA degradation (Fig. 1C).

**Figure 1:**
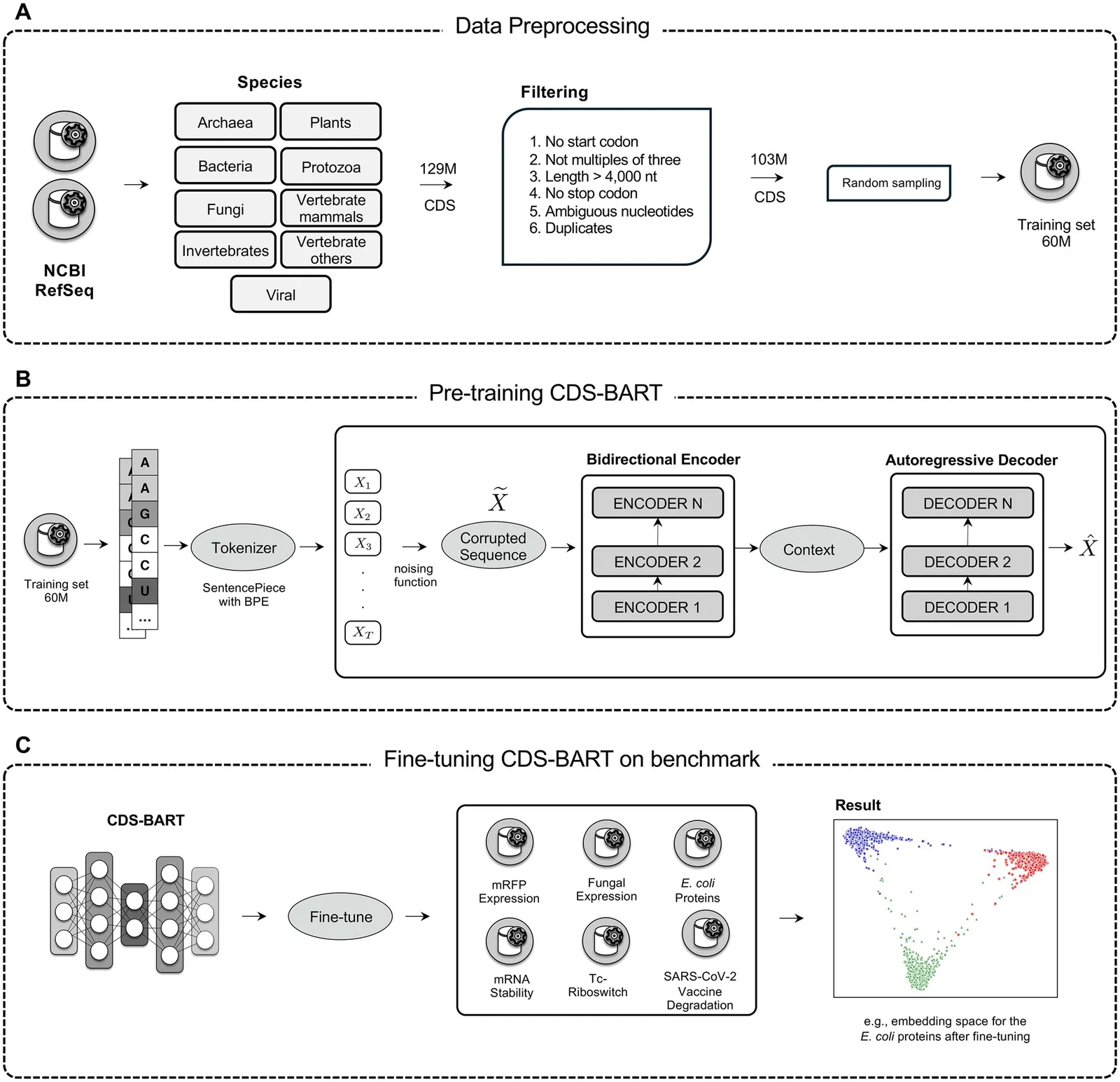
The overall procedure for establishing CDS-BART. (A) In the data collection and processing step, we assembled a training dataset from the NCBI RefSeq database. This process involves the extraction of mRNA sequences from diverse organisms categorized in nine taxonomic groups. (B) CDS-BART was trained to learn mRNA sequence context by corrupting sequences with noising functions and reconstructing the original sequences. (C) To evaluate the performance of CDS-BART, we fine-tuned our models on six different mRNA tasks (Table S1). The labeled datasets were split into training, validation, and test sets in proportions of 70%, 15%, and 15%, respectively.

### 2.2 Dataset for pre-training

To assemble a comprehensive training dataset for pre-training, we collected mRNA coding sequences from the NCBI RefSeq database (Goldfarb *et al.* 2025). The NCBI RefSeq database categorizes organisms into nine taxonomic groups, including archaea, bacteria, fungi, invertebrates, plants, protozoa, vertebrate mammals, other vertebrate, and viral species. Using the genome assembly summary file, we downloaded the coding sequences (CDSs) of the representative and reference genomes belonging to the nine taxonomic groups via the NCBI FTP site. The composition of CDS sequences by taxonomic group is shown in Fig. S1. After applying stringent filtering criteria and removing duplicates, we obtained 103 million unique CDSs, from which we randomly sampled about 60 million for pre-training. Filtering details are provided in the Supplementary Methods. The overall dataset preprocessing workflow is illustrated in Fig. 1A.

### 2.3 Tokenizer training

To process longer sequences without increasing the model size, we developed a novel mRNA CDS tokenizer that represents sequences as recurrent units of varying lengths based on their frequency in the training dataset. Then, we tokenized the input sequences using SentencePiece, which treats each CDS sequence as a continuous stream without requiring predefined word-like boundaries. The tokenizer was trained using byte-pair encoding (BPE), which iteratively merges frequent adjacent symbols to construct variable-length tokens from the corpus. In contrast to fixed nucleotide-level or codon-level tokenization, the resulting token boundaries are learned from corpus-level sequence regularities rather than being imposed at every nucleotide or every triplet. Thus, a learned token may correspond to a sub-codon fragment, a codon-scale unit, or a longer recurrent sequence pattern depending on its frequency in the CDS corpus. We trained the tokenizer using BPE with a vocabulary size of 4096 to process 60 million CDSs. The tokenization process required approximately 4.3 TB of memory and took two days, seven hours, and 55 minutes until completion. The trained SentencePiece-BPE tokenizer was subsequently applied to each CDS to generate variable-length token IDs. Further implementation details are provided in the Supplementary Methods (“1–3. Tokenizer training”).

### 2.4 Model pre-training

CDS-BART is a BART-based denoising autoencoder built on a seq2seq architecture (Fig. 1B). The model comprises two main components: a bidirectional encoder and an autoregressive decoder (Fig. S2). CDS-BART can learn the sequence context by corrupting sequences with arbitrary noising functions and reconstructing the original sequences (Fig. S3). For pre-training, we employed the BART transformer architecture, consisting of 12 encoder and decoder layers, 12 attention heads, and an embedding dimension of 768. We padded or truncated CDS sequences to a maximum length of 850 tokens.

We trained three model variants independently, each employing a distinct pre-training objective: CDS-BART-seq2seq, CDS-BART-CLM, and CDS-BART-denoising. These variants were not conducted jointly; each variant was trained separately and evaluated on the mRFP Expression dataset to select the best-performing pre-training approach. CDS-BART-seq2seq uses a standard transformer encoder-decoder architecture, while CDS-BART-CLM utilizes a decoder-only architecture for next-token prediction. Finally, CDS-BART-denoising employs a random token masking strategy in which 10% of the input tokens in each CDS sequence are randomly selected and replaced with a special mask token before encoding. The encoder processes this corrupted sequence, and the decoder is trained to autoregressively reconstruct the original, uncorrupted token sequence. We used random token masking as the noising function because CDS sequences lack natural sentence-like boundaries, making span- or permutation-based noise less appropriate than for natural language. This denoising objective was designed to encourage robust contextual learning across CDS tokens by allowing the model to use bidirectional context during encoding while retaining an explicit reconstruction objective. All models were trained using the AdamW (Loshchilov and Hutter 2019) optimizer with a learning rate of 5e-5, a weight decay of 0.01, and a linear learning rate warmup ratio of 0.1. Our model training was performed on 8 NVIDIA A100 GPUs using the Hugging Face (Wolf *et al.* 2020) and DeepSpeed (Rajbhandari *et al.* 2020) accelerator libraries.

### 2.5 Model fine-tuning

For fine-tuning, we used six out of the seven benchmark datasets provided by CodonBERT, excluding the MLOS Flu Vaccine dataset because the metadata of this dataset did not match the description in the CodonBERT paper. The six benchmark datasets were mRFP Expression, *Escherichia coli* Proteins, mRNA Stability, Tc-Riboswitches, Fungal Expression, and SARS-CoV-2 Vaccine Degradation. These datasets cover diverse mRNA-related prediction tasks, including protein production, protein expression classification, mRNA stability, riboswitch switching factor, fungal gene expression, and RNA degradation. *Escherichia coli* Proteins was formulated as a three-class classification task, whereas the remaining five datasets were formulated as regression tasks. We truncated the sequences of Fungal Expression and mRNA Stability to maximum lengths of 3000 and 1497 nucleotides, respectively. Then, we split the train, validation, and test data according to the labeled benchmark datasets (Table S1). Fine-tuning was conducted on a single NVIDIA A100 (80 GB) GPU using the Hugging Face library. The pre-trained CDS-BART checkpoint was fine-tuned for sequence-level prediction, with task-specific output dimensions for regression or classification. Hyperparameters were empirically optimized for each task, as detailed in Table S2. Regression tasks were evaluated using Spearman’s rank correlation, whereas the *E. coli* Proteins classification task was evaluated using accuracy.

### 2.6 Software utility and availability

CDS-BART was designed as a practical and reusable framework for CDS-based mRNA sequence analysis. The released implementation provides a pre-trained CDS-BART checkpoint, tokenizer, benchmark datasets, and experiment-specific configuration files for six downstream tasks. Users can fine-tune the model on regression or classification tasks by specifying the dataset, label column, maximum input length, and task type through Hydra configuration files. The fine-tuning script loads datasets through the Hugging Face datasets library, tokenizes the seq field using BartTokenizerFast, dynamically pads batches, and fine-tunes BartForSequenceClassification using the Hugging Face Trainer. This design allows CDS-BART to be reused for new mRNA property prediction tasks with minimal changes to the dataset and configuration files.

## 3 Results

First, we evaluated pre-training objectives for mRNA sequence property prediction (Fig. 1C). We found that the CDS-BART-denoising model outperformed the other two models (CDS-BART-CLM and CDS-BART-seq2seq), achieving a Spearman correlation of 0.88 with the mRFP Expression dataset (Fig. S4). This performance underscores the effectiveness of the corruption-denoising strategy (Fig. S3), which appears to capture robust mRNA sequence patterns during training.

Next, we compared CDS-BART-denoising (hereafter referred to as CDS-BART) against other published methods, including CodonBERT (Li *et al.* 2024), TextCNN (Kim 2014), RNABERT (Akiyama and Sakakibara 2022), RNA-FM (Chen *et al.* 2022) and TF-IDF (Rajaraman and Ullman 2011) in Table 1.

**Table 1:** Results of the evaluation on classification/regression of the benchmark datasets.^a^

Model	mRFP Expression	*Escherichia coli* proteins	mRNA stability	Tc-Riboswitch	Fungal expression	SARS-CoV-2 vaccine degradation
Dataset task	Regression	Classification	Regression	Regression	Regression	Regression
**Nucleotide-based**						
**plain TextCNN**	0.62	0.39	0.01	0.41	0.53	0.55
**RNABERT + TextCNN**	0.4	0.39	0.16	0.47	0.41	0.64
**RNA-FM + TextCNN**	0.8	0.43	0.34	0.58	0.59	0.74
**Codon-based**						
**TF-IDF**	0.57	0.44	0.54	0.49	0.68	0.69
**plain TextCNN**	0.78	0.36	0.26	0.43	0.76	0.8
**Codon2vec + TextCNN**	0.77	0.43	0.33	0.56	0.61	0.7
**CodonBERT**	0.85	0.55	0.51	0.56	**0.88**	0.77
**CDS-BART**	**0.88**	**0.57**	**0.56**	**0.66**	0.82	**0.86**

a Comparison of CDS-BART to prior methods on six downstream tasks. For regression tasks, the corresponding Spearman’s rank correlation values were used. For the classification task (*E. coli* proteins dataset), classification accuracy was calculated. The best value of correlation and accuracy for each task was marked in bold.

When compared to other methods, CDS-BART demonstrated notable strengths. In particular, the SARS-CoV-2 Vaccine Degradation and Tc-Riboswitch tasks are critical for understanding and improving the stability and efficacy of RNA therapeutics. In the evaluation of these tasks, CDS-BART surpassed CodonBERT, achieving remarkable improvements of 11.69% and 17.86%, respectively.

As summarized in Table 1 (and visualized in Fig. S5), CDS-BART outperformed CodonBERT on five of six benchmarks, demonstrating robust performance overall (Fig. S6). CDS-BART’s performance was slightly lower than CodonBERT in the Fungal Expression task (0.82 vs. 0.88). The dataset includes many fungal species with experimentally measured expression levels (Wint *et al.* 2022), and shows a multi-modal label distribution (Fig. S7), potentially reflecting strong codon-usage bias and GC-content variability among taxa. As reported in CodonBERT’s benchmarks, codon-level inputs improved performance on several downstream tasks compared with nucleotide-level inputs under the same architecture (Li *et al.* 2024). In our comparative analysis, CodonBERT’s codon-based embeddings appeared to capture fungal translation patterns slightly better. However, CDS-BART’s token-based embeddings demonstrated higher performance across a broader range of tasks, supporting its utility as a general CDS-level representation model for mRNA property prediction.

To further evaluate CDS-BART on long coding sequences, we constructed a non-overlapping GenBank-derived CDS evaluation panel and a length-stratified long-sequence benchmark spanning <1 kb to 4–5 kb. We fine-tuned CDS-BART to predict standard-scaled minimum free energy (MFE) and evaluated performance across length bins. This analysis provides an additional assessment of CDS-BART’s behavior on long CDSs, including sequences around and above 4 kb. It shows how BPE token counts relate to the 850-token context limit (Supplementary Methods, Fig. S8). We also performed perturbation and probing analyses to characterize whether CDS-BART representations capture CDS-related regularities. Mutational trajectory and likelihood analyses examined synthetic synonymous, missense, nonsense, and frameshift mutations, whereas linear probes evaluated motif-context and coding-constraint information in frozen encoder representations. These analyses suggest sensitivity to disruptive perturbations and ORF-boundary-related contexts (Fig. S9). However, diagnostic probes did not provide strong evidence for explicit codon-boundary or reading-frame encoding, and disease-associated SNP analysis did not show a significant likelihood difference between ClinVar benign and pathogenic variants (Fig. S10).

## 4 Discussion

In this study, we present CDS-BART, a BART-based mRNA foundation model that can extend the input sequence limit to about 4 kb, corresponding to the typical payload size of many LNP-delivered therapeutic mRNAs. CDS-BART consistently achieved high accuracy across the six benchmark tasks except for the Fungal Expression task, which is characterized by the multimodal data distribution of multiple species. The modest difference in the Fungal Expression task likely reflects representation effects, where CodonBERT’s explicit codon embeddings capture fungal-specific translation biases more directly than CDS-BART’s generalized subword embeddings. Our model also demonstrated improved results compared to CodonBERT in the SARS-CoV-2 Vaccine Degradation and Tc-Riboswitch tasks, which may benefit from contextual sequence representations learned through pre-training and the bidirectional-plus-autoregressive architecture. These properties are well suited to the token-masking denoising and long-context nature of therapeutic mRNA sequence modeling. Moreover, CDS-BART is released as an easy-to-use and openly licensed toolkit, whose pre-trained and fine-tuned weights are publicly available on GitHub and Hugging Face. To support reproducibility and transparency, the codebase has also been archived on Zenodo with a citable DOI (10.5281/zenodo.21502775). This can lower the barrier for researchers in basic biology and mRNA-vaccine engineering to use AI-based sequence models.

On the other hand, the efficient utilization of computational resources is also critical to the development of mRNA foundation models. CDS-BART inherits the heavy computational footprint of a full encoder–decoder transformer. In detail, the dual self-attention blocks require approximately twice the memory of an encoder-only model such as BERT. We therefore limited the input length to 850 tokens in the current implementation. Although position-encoding methods such as ALiBi and RoPE can support extrapolation beyond the training sequence length, they do not eliminate the scaling costs of standard self-attention (Press *et al.* 2022, Su *et al.* 2024). Applying low-bit quantization such as Qrazor and reasoning-aware distillation like DeepSeek-R1’s approach (Guo *et al.* 2025, Lee *et al.* 2025) can make CDS-BART slimmer while enabling larger context windows for longer CDSs or full RNA sequences containing CDS and UTRs together. In addition, because the same encoder-decoder backbone already powers strong text generation, a compressed CDS-BART could auto-complete missing segments or design full transcripts de novo. Such capability is expected to support downstream wet-lab validation for cellular protein expression and molecular stability. Although CDS-BART shows sensitivity to disruptive perturbations and ORF-boundary-related sequence contexts, our diagnostic analyses did not support a strong claim that the current model explicitly encodes codon-boundary or reading-frame structure. In addition, ClinVar benign and pathogenic variants did not show a significant likelihood difference, indicating that CDS-BART should not be interpreted as a pathogenicity predictor without task-specific fine-tuning. We therefore interpret CDS-BART as a CDS-aware sequence representation model that captures corpus-level CDS regularities, rather than as an explicitly reading-frame-aware or biological motif-discovery model.

Taken together, these features support the use of CDS-BART as a practical and reusable tool for CDS-level mRNA sequence analysis, with potential applications in mRNA research, vaccine design, and therapeutic development.

## Supplementary Material

vbag259_Supplementary_Data

## Data Availability

The data underlying this article are available in Zenodo at https://doi.org/10.5281/zenodo.21502775. The source code is available on GitHub at https://github.com/mogam-ai/CDS-BART and is released under the MIT License.
