# Supplementary material for: CDS-BART: a BART-based foundation model for mRNA sequence analysis
Source: Bioinform Adv. 2026 Sep 15;6(1):vbag259. doi: 10.1093/bioadv/vbag259 (PMC13695146; doi:10.1093/bioadv/vbag259)
Supplement: Supplementary file 1 [file vbag259_supplementary_data.docx]

Supplementary Material for “CDS-BART: A BART-Based Foundation Model for mRNA Sequence Analysis” 
Erkhembayar Jadamba 1†, Sang-Heon Lee 1†, Sungho Lee 1, Hyekyoung Lee 1, Jinhee Hong 1, Hyunjin Shin 1*
1 MOGAM Institute for Biomedical Research, Seoul, 06730, Republic of Korea 
*Corresponding author. MOGAM Institute for Biomedical Research, Seoul, 06730, Republic of Korea. E-mail: hyunjin.shin@mogam.re.kr 
†Equal contribution.


Supplementary Methods
Data collection
To assemble a comprehensive training dataset for pre-training, we collected mRNA sequences of a variety of organisms from the NCBI RefSeq database [1]. The assembly summary file was accessed via [NCBI Assembly Summary] (https://ftp.ncbi.nlm.nih.gov/genomes/refseq/assembly_summary_refseq.txt), and genomic sequences were downloaded from the NCBI FTP site at [NCBI Genomes] (https://ftp.ncbi.nlm.nih.gov/genomes/all/). Our data extraction process focused on obtaining all coding sequences (CDS) of representative and reference genomes. The NCBI RefSeq database categorizes organisms into nine taxonomic groups, including archaea, bacteria, fungi, invertebrates, plants, protozoa, vertebrate mammals, vertebrate other, and viral species. This classification enabled us to compile a diverse dataset comprising a total of 35,694 species, ultimately resulting in the collection of 129 million CDS (Supplementary Figure 1).

Data processing
To ensure the integrity and relevance of the mRNA sequences for subsequent analyses, we implemented a rigorous preprocessing protocol that involved stringent filtering criteria. Specifically, we excluded sequences that did not begin with a start codon (ATG), those whose lengths were not multiples of three, sequences exceeding 4 kilobases (4 kb) in length, sequences lacking stop codons (TAG, TGA, TAA), and sequences containing ambiguous nucleotides (other than A, T, G, or C). Following the application of these criteria and the removal of duplicate sequences, we finally obtained a dataset of 103 million unique CDS, which served as the foundation for further analyses. For the pre-training datasets, we randomly selected 60 million raw coding DNA sequences (CDS) for the pre-training of the CDS-BART model.
To motivate the 4 kb length cutoff, we examined the length distribution of human CDSs separately. From the human RefSeq GRCh38.p14 annotation, 83,842 unique CDSs remained after applying the same integrity filters (excluding the length criterion) and removing duplicate sequences. These sequences averaged 1,757 nt in length, and 4,030 of them (4.81%) exceeded 4 kb.

Tokenizer training
To overcome the challenge of processing longer sequences without enlarging the model, we created a novel mRNA CDS tokenizer that identifies motifs of varying lengths, informed by their frequency in the training dataset. We utilized the SentencePiece [2] method, which treats the input sequence as a continuous stream without requiring predefined word-like boundaries, effectively tokenizing the input into variable-length subword units. SentencePiece operates as an unsupervised text tokenization framework that employs two subword algorithms: the Unigram language model and byte-pair encoding (BPE). The vocabulary size is established prior to program execution; hence, we trained the tokenizer using BPE with a vocabulary size of 4,096 [3]. To facilitate the training of 60 million CDS, we divided the data into chunks of 2 million sequences each. The process of training the tokenizer required approximately 4.3 TB of memory and took a total of 2 days, 7 hours, and 55 minutes to complete.

Model architecture
CDS-BART is a BART-based[4] denoising autoencoder built with a sequence-to-sequence model. 
As shown in Supplementary Figure 2, the CDS-BART architecture consists of two main components:
1. Encoder: A bidirectional encoder, like BERT, that processes the entire input sequence to understand the context of the text in both directions.
2. Decoder: An autoregressive decoder, like GPT [5] (Generative Pre-Training), that generates text sequentially, one token at a time, using the encoded representations.
The CDS-BART model is trained by corrupting a sequence with an arbitrary noising function and learning to reconstruct the original sequence (Supplementary Figure 3):
1. Corruption: The input CDS sequences are corrupted using a noising function as masking. We deliberately masked 10% of the input tokens in each sequence.
2. Reconstructions: The CDS-BART model learns to reconstruct the original, uncorrupted sequences from the noisy (masked) input.
The pre-training of CDS-BART on mRNA sequences starts with tokenizing raw sequences,  
$X=(X_{1}, X_{2}, \ldots, X_{T}$)                                 (1)
where $X_{j}$represents individual tokens, and T is the sequence length. Then CDS-BART applies a noising function, to X, producing a corrupted sequence $\tilde{X}$. We used masking of input sequences as our default noising function,  
$f_{noise}\left( X \right)= \tilde{X}.$(2)
The corrupted sequence $\tilde{X}$ is fed into the bidirectional encoder, which maps H to contextual embeddings:
$H=Encoder\left( \tilde{X} \right). (3)$
Here, $H \in\mathbb{R}^{Txd}$ , where d is the hidden size of the encoder, and each $h_{t}$ (a row of H) represents the contextualized embedding for the token $X_{t}$. 
The training objective is to maximize the log likelihood of reconstructing the original sequence X from the corrupted input $\tilde{X}$:
$\mathcal{L=}\mathbb{E}_{X\sim\mathcal{D}} \left[ log P\left( X \right|\tilde{X}) \right]$                             (4)

Where D is the training dataset. The probability is computed as:
$P\left( X \right|\tilde{X})= \prod_{t=1}^{T} P\left( x_{t} \right| x_{<t}, H)$                           (5)

Pre-training detail
We employed a BART transformer architecture for the seq2seq and denoising pre-training experiments, and a decoder-only transformer for the CLM variant. The base configuration consists of 12 encoder and decoder layers, 12 attention heads, an embedding dimension of 768. CDS sequences were padded or truncated to a maximum length of 850 tokens.
We pre-trained three models using distinct objectives.
seq2seq (Sequence-to-sequence (Seq2Seq) autoencoder). This model was trained as a standard transformer encoder-decoder. The input to the encoder was the complete, unprocessed CDS sequence (prepended with [<bos>] and appended with [<eos>]). The decoder was tasked with autoregressively generating the identical sequence, conditioned on the encoder's output and the previously generated tokens. The objective was to minimize the cross-entropy loss between the decoder's output logits and the target sequence.
CDS-BART-CLM (Causal Language Model). For the CDS-BART-CLM, we utilized a decoder-only transformer architecture. The model was trained to predict the next nucleotide token given the preceding tokens in the sequence. The objective was to minimize the standard cross-entropy loss for next-token prediction across the entire sequence.
CDS-BART-denoising (Denoising Seq2Seq). For this model we used the same transformer encoder-decoder as seq2seq. However, the input CDS fed to the encoder was corrupted using a masking strategy. Specifically, 10% of the input tokens in each sequence were randomly selected and replaced with the mask token.
All models were trained using the AdamW[6] optimizer with a learning rate of 5e-5, and weight decay 0.01. We employed a linear learning rate warmup ratio of 0.1. A batch size of 16 was used for the training and validation data. The pre-training process was performed on 8 x NVIDIA A100 (80GB) GPUs using Hugging Face [7] and DeepSpeed [8] accelerator library.
In addition, we assessed three different pre-training objectives, CDS-BART-CLM, CDS-BART-seq2seq, and CDS-BART-denoising, to investigate the relative advantages of these objectives in fine-tuning. The pre-training model variants were evaluated with the mRFP Expression dataset, yielding Spearman correlation of 0.640, 0.798, and 0.875, respectively. The results suggest that the higher performance of CDS-BART-denoising is likely due to its corruption-based pre-training strategy, which encourages the model to learn more robust contextual representations by recovering the corrupted tokens. This form of denoising forces the encoder to capture richer and more robust bidirectional dependencies within mRNA sequences, translating well to fine-tuning scenarios. In contrast, CDS-BART-seq2seq a seq2seq autoencoder, rather focuses on simply reconstructing the input, which may offer less representational challenges and diversity for homogeneous mRNA sequences. Lastly, CDS-BART-CLM, the decoder-only CLM, although known to be effective for sequence generation based on left-to-right context, may not be optimal for such tasks as requiring a global understanding of long mRNA sequences. These findings highlight the efficacy of the denoising pre-training strategy in enhancing the model's ability to generalize across different mRNA-related tasks.

Fine-tuning dataset
CodonBERT provides seven types of datasets, including MLOS Flu Vaccines, mRFP Expression [9], E. coli Proteins [10], mRNA Stability [11], Tc-Riboswitches [12], Fungal Expression [13], and SARS-CoV-2 Vaccine Degradation [14], which encompass diverse biological data that form a robust foundation for analyzing gene expression and protein production across various organisms and conditions. In our study, the MLOS Flu Vaccine dataset [15] was excluded due to discrepancies in the number of data points compared to the original paper and the lack of division into training and validation sets, resulting in the utilization of six out of the seven available datasets. Furthermore, the Fungal Expression and mRNA Stability datasets contained more data points than reported in the original paper; therefore, we truncated the sequences to maximum lengths of 3,000 and 1,497 nucleotides, respectively, to ensure consistency with CodonBERT. Subsequently, we employed the labeled datasets to perform the split into training, validation, and test sets, adhering to proportions of 70%, 15%, and 15%, respectively.

Fine-tuning detail
In our fine-tuning experiments, we utilized the Hugging Face library, leveraging a pre-trained BART transformer model. The fine-tuning process was conducted on a single NVIDIA A100 (80GB) GPU and aimed to optimize performance across six distinct tasks, as summarized in the accompanying fine-tuning dataset (Supplementary table 1). The following hyperparameters were selected based on empirical evaluations to achieve optimal performance on the respective tasks. The detailed hyperparameters for the best-performing models across each task are shown in the Supplementary Table 2, which provides a comprehensive overview of the configurations employed for each dataset.

Embedding space visualization details
To evaluate the effectiveness of the pre-trained model in capturing sequence patterns across different taxonomic groups, we encoded the data and projected it into a two-dimensional space to analyze clustering behavior relative to taxonomic classification. For this study, we selected four taxonomic groups plant, bacteria, invertebrate, and fungi from a total of nine groups, randomly sampling 500 CDS sequences from each. These sampled sequences were processed using the CDS-BART model to generate embeddings. Subsequently, we employed Principal Component Analysis (PCA) to reduce the dimensionality from 768 features to 50 features, facilitating more efficient data handling. Following this, Uniform Manifold Approximation and Projection (UMAP) was applied for projection, allowing for visualization of the clustering patterns. This approach revealed that sequences belonging to the same taxonomic group clustered together, delineating distinct boundaries, as illustrated in Supplementary Figure 11A.

2. Supplementary Methods for Additional Benchmark and Characterization Analyses 
Data collection and preprocessing for the non-overlapping CDS evaluation panel 
To evaluate generalization beyond the CDS-BART pre-training corpus, we constructed a non-overlapping coding sequence evaluation dataset from GenBank assemblies that were not included in the pre-training corpus. The CDS-BART pre-training corpus was based on RefSeq representative and reference genome CDS sequences, comprising approximately 60 million sequences. To minimize overlap with the pre-training data, we selected GenBank assemblies that did not have corresponding RefSeq representative or reference genome pairs. Candidate assemblies were filtered based on assembly quality and taxonomic diversity. Specifically, only assemblies at the Chromosome or Complete Genome level were retained. Assemblies showing evidence of contamination were excluded using thresholds of fraction_contaminated < 0.01 and total_contaminant_length < 100,000 bp. To avoid overrepresentation of specific taxa, the number of assemblies was capped at 200 per taxonomic group, with the exception of invertebrates, which were capped at 2,000 assemblies.
To further remove potential overlap with the pre-training corpus, exact sequence deduplication was performed against the pre-training dataset using a pre-built binary index based on a C++ exact-match hash implementation. CDS sequences were then subjected to basic quality control. We retained only sequences with valid start and stop codons, lengths divisible by three, no internal in-frame stop codons, and ACGT-only nucleotide composition. The resulting balanced evaluation panel consisted of 45,000 CDS sequences distributed across nine taxonomic groups and four length bins: <1 kb, 1–2 kb, 2–3 kb, and 3–4 kb. Assembly-level and taxonomic group-level caps were applied throughout sampling to prevent overrepresentation by any single assembly or group.

Long-sequence benchmark dataset 
For length-stratified analyses, we generated an extended long-sequence benchmark dataset from the full non-overlapping CDS pool. This dataset consisted of 5,000 CDS sequences distributed across five length bins: <1 kb, 1–2 kb, 2–3 kb, 3–4 kb, and 4–5 kb, with 1,000 sequences sampled from each bin. To maintain diversity and reduce redundancy, sampling was constrained using a per-assembly cap of 20 sequences per bin and a maximum taxonomic group fraction of 35% within each bin.

Motif probe window generation 
To evaluate whether CDS-BART representations encode motif-discriminative sequence contexts, we generated motif probe windows from 3,000 MANE Select human transcripts. Each motif window was 63 nt in length, corresponding to 21 codons, and was centered on the target codon with ±10 codons of flanking context. For each transcript, twelve motif types were generated, including positive windows representing start codons, stop codons, downstream regions of start codons, and upstream regions of stop codons, as well as matched negative windows representing GC-matched background contexts, internal start-like contexts, induced stop-codon contexts, and terminal-proximal controls.
Negative windows were matched to their corresponding positive windows by GC content and sampled from the same transcript to minimize confounding by transcript-level sequence composition. Windows located within ±15 nt of true start or stop codons were avoided when constructing background controls. Approximately 3,000 windows were generated for each motif type, yielding a total of approximately 36,000 motif windows. This paired and GC-matched design was intended to assess motif-specific representation while controlling for local sequence composition and transcript identity.

Perturbation scoring framework 
All perturbation-based analyses were performed using a paired wild-type and mutant scoring framework. For each wild-type/mutant pair, the likelihood penalty was defined as the negative difference between the mutant and wild-type log probabilities. Positive values indicated that the model assigned a lower probability to the mutant sequence than to the corresponding wild-type sequence. In addition to full-sequence likelihood scoring, we computed affected-token ΔNLL, defined as the mean negative log-likelihood difference restricted to BPE tokens overlapping the mutation site. Local-window scoring was also performed using a 301-nt sequence window centered on each mutation. Sanity checks were applied to confirm that the variant was located within the window and that the local wild-type and mutant sequences differed at the expected position.

Mutational trajectory analysis
To examine how different classes of mutations affect the learned embedding space, we generated synthetic mutations from 5,000 sampled CDS sequences. Four mutation classes were considered: synonymous substitutions, missense substitutions, nonsense substitutions, and frameshift mutations. Synonymous mutations were defined as single-nucleotide substitutions that preserved amino acid identity, whereas missense mutations changed the encoded amino acid. Nonsense mutations introduced premature stop codons, and frameshift mutations were generated by introducing a 1-bp deletion in the middle of the CDS. Approximately 4,900 events were generated for each mutation class.
For each wild-type and mutant sequence pair, encoder hidden-state embeddings were extracted from the last layer and mean-pooled across the sequence. All embeddings corresponding to 19,592 synthetic mutation events were projected into a two-dimensional space using principal component analysis. Mutational displacement was quantified as the Euclidean distance between wild-type and mutant embeddings in the resulting principal-component space.

Frameshift recovery
To evaluate the model’s sensitivity to reading-frame disruption and potential recovery from shifted sequence contexts, we generated +1 and +2 frameshift perturbations from 1,000 CDS sequences. Frameshifts were introduced by deleting nucleotides at two distinct anchor positions: the middle of the CDS and immediately after the start codon, corresponding to position 4. For each anchor mode, 2,000 frameshift events were generated. This setup enabled assessment of whether CDS-BART detects and penalizes frame-disrupting perturbations consistently across different positions within coding sequences.

Codon-boundary effect analysis
To assess whether CDS-BART captures codon-boundary structure, we generated dinucleotide substitutions at both codon-internal and codon-boundary-crossing positions. A total of 3,000 events were generated for each condition. Sampling was balanced across codon positions and relative positions within the CDS to minimize positional confounding. By comparing model responses to codon-internal and boundary-crossing substitutions, this analysis tested whether the model assigns differential importance to perturbations that disrupt sequence structure across codon boundaries.

Disease-associated SNP analysis
To evaluate whether CDS-BART scores reflect disease relevance, we analyzed ClinVar germline single-nucleotide variants annotated as pathogenic or likely pathogenic and benign or likely benign. Variants were mapped to MANE transcripts and scored using sequence windows centered on the variant position. The window size was set to 101 nt. To control for gene-level confounding, pathogenic and benign variants were sampled from the same shared gene pool, comprising 4,376 genes represented in both clinical classes. For each class, 1,000 variants were scored. This gene-matched design allowed comparison of model-derived penalties between clinically deleterious and benign variants while reducing bias due to differences in gene identity.

Motif representation probes 
To examine whether motif information is encoded in the hidden representations of CDS-BART, frozen encoder hidden states were extracted for each 63-nt motif window. Mean-pooled hidden states across sequence positions were computed for each encoder layer, covering layers 0 through 12. Binary logistic regression probes were trained across ten comparison conditions, including start_codon versus internal_start_context and stop_upstream versus induced_stop_upstream. To prevent information leakage across related examples, train, validation, and test splits were performed at the transcript level using a 70/15/15 split. Probe performance was evaluated using AUROC on the held-out test set. This analysis was designed to identify which layers encode motif-discriminative information and whether biologically meaningful sequence contexts are linearly decodable from the pre-trained representations.

Coding-constraint probes 
To evaluate whether CDS-BART encodes position-level coding constraints, we trained binary probes using nucleotide-level hidden representations. For each sampled CDS sequence, token-level encoder hidden states were projected back to nucleotide positions and used as feature vectors. Binary labels included wobble-position status, synonymous substitutability, and nonsense sensitivity. The probe dataset consisted of approximately 2,540 transcripts with 300 sampled positions per transcript, resulting in approximately 762,000 total nucleotide-level samples. This analysis assessed whether the pre-trained model captures constraints associated with codon position, mutational tolerance, and stop-codon sensitivity at single-nucleotide resolution.

Reading-frame probes 
To evaluate whether reading-frame information is encoded in CDS-BART representations, we trained probes for both position-level and sequence-level frame-related tasks. Position-level probes were trained for three-class phase classification, corresponding to codon phases 0, 1, and 2, and for binary codon-boundary detection. These analyses used projected hidden states from 500 transcripts with 50 sampled positions per transcript, yielding 25,000 position-level samples. In addition, a sequence-level frame-state probe was trained to distinguish in-frame, +1-shifted, and +2-shifted sequences using mean-pooled sequence embeddings from 500 transcripts across three frame conditions, yielding 1,500 samples. Multiclass AUROC was computed using one-vs-rest averaging. Together, these analyses examined whether CDS-BART representations encode both local codon-phase information and global reading-frame disruption.

MFE fine-tuning task setup 
For the MFE fine-tuning benchmark, CDS-BART was fine-tuned to predict standard-scaled MFE values from RNA sequence input. The dataset consisted of 5,000 CDS sequences sampled across five length bins, with 1,000 sequences per bin. Minimum free energy values were computed for all 5,000 CDS sequences in the long-sequence benchmark dataset using ViennaRNA. Prior to folding, DNA sequences were converted to RNA sequences by replacing thymine with uracil. RNA folding was performed using RNA.fold_compound followed by fc.mfe(). Raw MFE values, expressed in kcal/mol, were recorded.
The dataset was split into training, validation, and test sets using a 3,500/750/750 split, with stratification by length bin. The test set contained 150 sequences from each length bin. The prediction target was standard-scaled MFE, enabling evaluation of whether the pre-trained sequence model could be adapted to a continuous RNA structural-property prediction task.

MFE fine-tuning model and training 
For MFE prediction, BartForSequenceClassification with num_labels=1 was initialized from the pre-trained CDS-BART-denoising checkpoint. The model was fine-tuned using a learning rate of 2×10⁻⁵, a cosine learning-rate scheduler, a warmup ratio of 0.1, weight decay of 0.01, and a batch size of 32 with gradient accumulation over four steps, corresponding to an effective batch size of 128. Training was conducted for 40 epochs. Standard scaling (zero mean, unit variance) was applied to the MFE target prior to fine-tuning. The maximum token length was set to 850, and sequences exceeding this length were truncated. The best-performing model was selected based on validation loss, with the optimal checkpoint obtained at epoch 18.

MFE fine-tuning evaluation
The fine-tuned MFE prediction model was evaluated on the held-out test set using Spearman correlation. Performance was computed both globally across the full test set and separately within each length bin. This evaluation strategy allowed assessment of overall predictive performance as well as robustness across sequence-length strata. The final reported results correspond to the checkpoint selected by validation loss, ensuring that test-set performance reflected model generalization rather than post hoc model selection.


Supplementary Figures
Supplementary Figure 1. NCBI raw data taxonomy distribution used for pre-training. 
Alt text: A pie chart illustrates the taxonomic distribution of NCBI raw data, with colored slices representing the biological groups used for pre-training.


Supplementary Figure 2. CDS-BART bidirectional encoder and autoregressive decoder.
Alt text: A schematic diagram depicts the CDS-BART architecture, showing the bidirectional encoder and the autoregressive decoder and their connections.


Supplementary Figure 3. CDS-BART pre-training process with corruption and reconstruction
Alt text: A schematic diagram illustrates the CDS-BART pre-training process, showing input sequence corruption by a noising function and subsequent reconstruction.


Supplementary Figure 4. Comparing the results of three types of pre-training models CDS-BART-CLM, CDS-BART-seq2seq, CDS-BART-denoising specifically focusing on their performance on the mRFP Expression dataset.
Alt text: A bar chart compares the Spearman correlation of three pre-training objectives, CDS-BART-CLM, CDS-BART-seq2seq, and CDS-BART-denoising, on the mRFP Expression dataset.


Supplementary Figure 5. Comparison of CDS-BART with prior methods across six benchmark tasks.
Alt text: A grouped bar chart compares CDS-BART against prior methods across six benchmark tasks, showing Spearman correlation values for each method.


Supplementary Figure 6. Performance distribution of CDS-BART with five random seeds for each task. This distribution demonstrates robust and stable performance.
Alt text: A violin plot shows the distribution of CDS-BART performance across five random seeds for each benchmark task, indicating stability.

Supplementary Figure 7. Task-specific label distributions for prediction targets.
Alt text: Six histograms show the label value distributions of the prediction targets for the six benchmark tasks.


Supplementary Figure 8. Length-stratified MFE prediction benchmark using standard-scaled MFE. (A) Test-set Spearman correlation between predicted and observed standard-scaled MFE, stratified by sequence-length bin. Each bin contains 150 test sequences. (B) Scatter plot of predicted versus observed standard-scaled MFE for all 750 test sequences, colored by sequence-length bin. The dashed diagonal line indicates perfect agreement between predicted and observed values. (C) Absolute prediction residuals by sequence-length bin, shown as median with interquartile range (IQR). (D) BPE token count as a function of nucleotide length for all 5,000 sequences used in MFE fine-tuning. The dashed horizontal line indicates the maximum model context length of 850 tokens. Points are colored by truncation status.
Alt text: A multi-panel figure shows the length-stratified MFE prediction benchmark, with a bar chart of Spearman correlation by length bin and scatter plots of predicted versus observed values.


Supplementary Figure 9. Characterization of CDS-BART representations using mutation perturbations and linear probes. (A–C) Mutational trajectory analysis. (A) Principal component analysis (PCA) projection of encoder representations for wild-type (WT; grey) and mutant coding sequences. Arrows indicate WT-to-mutant displacement in representation space, and mutant sequences are colored by mutation class. Synthetic mutations were generated from 5,000 CDS sequences sampled from non-overlapping GenBank assemblies that were excluded from the CDS-BART pre-training corpus. (B) Median displacement in PCA space by mutation class, shown with interquartile ranges (IQRs). (C) Median likelihood penalty, defined as −Δlog-likelihood, by mutation class. The dotted horizontal line indicates zero, corresponding to no likelihood penalty. (D–I) Linear probing analyses of motif and coding-constraint representations. (D–H) Layer-wise AUROC values for binary linear probes trained on frozen encoder hidden states to distinguish 63-nt codon-aligned motif windows from matched negative controls: (D) start downstream versus background, (E) stop upstream versus background, (F) combined start/stop contexts versus background, (G) start context versus background, and (H) stop context versus background. For the motif probes, approximately 3,000 windows were generated per motif type, with transcript-level train, validation, and test splits of 70%, 15%, and 15%, respectively. (I) Layer-wise AUROC for the nonsense-sensitive position probe from the coding-constraint analysis. Dashed vertical lines indicate the best-performing layer for each probe, and dotted horizontal lines indicate chance performance (AUROC = 0.5).
Alt text: A multi-panel figure shows mutation perturbation and linear probe analyses, including PCA projections of embeddings and layer-wise AUROC values across tasks.


Supplementary Figure 10. Diagnostic analyses of perturbation penalties and representation probes. (A–C) Perturbation likelihood penalties, shown as medians with interquartile ranges (IQRs). Positive values indicate that the model assigned a lower likelihood to the perturbed sequence than to the corresponding wild-type sequence. (A) Frameshift recovery analysis stratified by anchor position and shift size. Four conditions are shown, corresponding to +1 and +2 shifts introduced either immediately after the start codon or in the middle of the CDS. (B) Codon-boundary effect analysis comparing codon-boundary-crossing and codon-internal dinucleotide substitutions. (C) Disease-associated SNP analysis using 101-nt local windows, comparing ClinVar pathogenic or likely pathogenic variants with benign or likely benign variants. (D) Layer-wise AUROC values for coding-constraint probes trained on frozen encoder hidden states. Probes were trained to predict wobble-position status, synonymous substitutability, and codon-position class. The dotted horizontal line indicates chance performance (AUROC = 0.5). (E) Layer-wise AUROC values for reading-frame probes trained on frozen encoder hidden states. Probes were trained for codon-boundary detection, reading-frame phase classification, and frame-state discrimination among in-frame, +1-shifted, and +2-shifted sequences. The dotted horizontal line indicates chance performance (AUROC = 0.5).
Alt text: A multi-panel figure shows perturbation likelihood penalties as medians with interquartile ranges and layer-wise AUROC curves for representation probes.


Supplementary Figure 11. CDS-BART embedding space representation.
Alt text: A multi-panel figure shows UMAP projections of CDS-BART embeddings colored by NCBI taxonomy and by E. coli protein expression level.
(A) The embedding space of the pre-training model for four NCBI taxonomies is represented using UMAP     


(B) The fine-tuned model’s embedding space for the E. coli proteins classification task is represented using UMAP. E. coli proteins are categorized into low, medium and high expression levels.

	


Supplementary Figure 12. Fine-tuning train/evaluation loss.
Alt text: A line graph shows the fine-tuning training and validation loss curves of CDS-BART across epochs.


Supplementary Tables
Supplementary Table 1.  The details of the benchmark dataset used in evaluation of the CDS-BART
Dataset	Target	Category	No. of mRNAs	Seq length
mRFP expression [9]	Expression	Regression	1459	678-678
E. coli proteins [10]	Expression	Classification	6,348	171-3000
mRNA stability [11]	Stability	Regression	41,063	30-1497
Tc-Riboswitches [12]	Switching factor	Regression	355	66-75
Fungal expression [13]	Expression	Regression	7,053	150-3000
SARS-CoV-2 vaccine degradation [14]	Degradation	Regression	2,400	81-81

Each dataset is split into training, validation, and test with a 0.7, 0.15, and 0.15 ratio. All the methods were optimized on the same data split.

Supplementary Table 2.  Optimal hyperparameter configurations across six benchmark datasets
Task	Learning rate	Batch size	Warmup ratio	Weight decay	Learning rate Scheduler	Epoch	Normalization
mRFP expression	2e-5	4	0.1	0.01	Linear	30	Standard Scaling
E. coli proteins	1e-5	64	0.1	0.01	Cosine	30	-
mRNA Stability	2e-5	128	0.1	0.01	Cosine	30	-
Tc-Riboswitch	1e-5	4	0.1	0.01	Linear	30	-
Fungal Expression	2e-5	64	0.1	0.01	Linear	30	-
SARS-CoV-2  Vaccine Degradation	1e-5	4	0.1	0.01	Linear	30	-


References
1.	Goldfarb T, Kodali VK, Pujar S et al. NCBI RefSeq: reference sequence standards through 25 years of curation and annotation, Nucleic Acids Res 2025;53:D243-D257. https://doi.org/10.1093/nar/gkae1038
2.	Kudo T, Richardson J. SentencePiece: A simple and language independent subword tokenizer and detokenizer for Neural Text Processing. In: Proceedings of the 2018 Conference on Empirical Methods in Natural Language Processing: System Demonstrations. Brussels, Belgium: Association for Computational Linguistics; 2018. p. 66-71. https://doi.org/10.18653/v1/d18-2012
3.	Zhou Z, Ji Y, Li W et al. DNABERT-2: Efficient Foundation Model and Benchmark for Multi-Species Genomes. In: The Twelfth International Conference on Learning Representations. Vienna, Austria: International Conference on Learning Representations; 2024.
4.	Lewis M, Liu Y, Goyal N et al. BART: Denoising Sequence-to-Sequence Pre-training for Natural Language Generation, Translation, and Comprehension. In: Proceedings of the 58th Annual Meeting of the Association for Computational Linguistics. Online: Association for Computational Linguistics; 2020. p. 7871-7880. https://doi.org/10.18653/v1/2020.acl-main.703
5.	Radford A, Narasimhan K, Salimans T et al. Improving Language Understanding by Generative Pre-Training. 2018.
6.	Loshchilov I, Hutter F. Decoupled Weight Decay Regularization. arXiv:1711.05101, https://doi.org/10.48550/arXiv.1711.05101, 2017, preprint: not peer reviewed.
7.	Wolf T, Debut L, Sanh V et al. Transformers: State-of-the-Art Natural Language Processing. In: Proceedings of the 2020 Conference on Empirical Methods in Natural Language Processing: System Demonstrations. Online: Association for Computational Linguistics; 2020. p. 38-45. https://doi.org/10.18653/v1/2020.emnlp-demos.6
8.	Rajbhandari S, Rasley J, Ruwase O et al. ZeRO: Memory Optimizations Toward Training Trillion Parameter Models. In: SC20: International Conference for High Performance Computing, Networking, Storage and Analysis. Atlanta, GA, USA: IEEE; 2020. p. 1-16. https://doi.org/10.1109/sc41405.2020.00024
9.	Nieuwkoop T, Terlouw BR, Stevens KG et al. Revealing determinants of translation efficiency via whole-gene codon randomization and machine learning, Nucleic Acids Res 2023;51:2363-2376. https://doi.org/10.1093/nar/gkad035
10.	Ding Z, Guan F, Xu G et al. MPEPE, a predictive approach to improve protein expression in E. coli based on deep learning, Comput Struct Biotechnol J 2022;20:1142-1153. https://doi.org/10.1016/j.csbj.2022.02.030
11.	Diez M, Medina-Muñoz SG, Castellano LA et al. iCodon customizes gene expression based on the codon composition, Sci Rep 2022;12:12126. https://doi.org/10.1038/s41598-022-15526-7
12.	Groher A-C, Jager S, Schneider C et al. Tuning the Performance of Synthetic Riboswitches using Machine Learning, ACS Synth Biol 2019;8:34-44. https://doi.org/10.1021/acssynbio.8b00207
13.	Wint R, Salamov A, Grigoriev IV. Kingdom-Wide Analysis of Fungal Protein-Coding and tRNA Genes Reveals Conserved Patterns of Adaptive Evolution, Mol Biol Evol 2022;39:msab372. https://doi.org/10.1093/molbev/msab372
14.	Wayment-Steele HK, Kladwang W, Watkins AM et al. Deep learning models for predicting RNA degradation via dual crowdsourcing, Nat Mach Intell 2022;4:1174-1184. https://doi.org/10.1038/s42256-022-00571-8
15.	Li S, Moayedpour S, Li R et al. CodonBERT large language model for mRNA vaccines, Genome Res 2024;34:1027-1035. https://doi.org/10.1101/gr.278870.123
